# Supplementary material for: The association between role model presence and self-regulation in early adolescence: A cross-sectional study
Source: PLoS One. 2019 Sep 19;14(9):e0222752. doi: 10.1371/journal.pone.0222752 (PMC6752835; doi:10.1371/journal.pone.0222752)
Supplement: S1 File — (PDF) [file pone.0222752.s001.pdf]

1. Role model presence

あなたが最も尊敬する人は誰ですか？　一人だけあげてください。

Who is the person you most look up to? Please write only one name of the person.

2. Hopefulness in future expectations

あなたは、これからの人生にどのくらい希望をもっていますか？

To what extent do you feel hopeful about the future of your life?

「希望をまったくもっていない」を 0 点、「希望にみちあふれている」を 10 点とすると、何点くらいになると思いますか？

Please answer your level of hopefulness between 0 (Not at all) and 10 (extremely).

希望をまったくもってない<br>Not at all	希望にみちあふれている<br>Extremely

0 — 1 — 2 — 3 — 4 — 5 — 6 — 7 — 8 — 9 —10

3. Self-regulation

3-1. Attitude toward work

次の３つのうち、お子さんに最も当てはまるのはどれですか。

Which statement in each group best describes this child?

1．とても勤勉である
A very hard worker
2．平均的な勤勉さである
Average-works moderately well
3．勤勉でなく、怠けている
A poor worker or lazy

3-2. Concentration

次の３つのうち、お子さんに最も当てはまるのはどれですか。

Which statement in each group best describes this child?

1．とても集中力がある
One with higher power of concentration
2．平均的な集中力である
Average-concentrates moderately well
3．ほとんど、または、まったく集中力がない
Little or no power of sustained concentration

3-3. Neatness in work

次の３つのうち、お子さんに最も当てはまるものはどれですか。

Which statement in each group best describes this child?

１．とてもまじめでしっかりしている
Extremely neat and tidy in work

２．平均的なまじめさで、ある程度しっかりしている
Average-moderately neat and tidy

３．とてもだらしがない
Very untidy in work

3-4. Daydreaming at home

次の３つのうち、お子さんに最も当てはまるものはどれですか。

Which statement in each group best describes this child?

１．ボーッとしていることはない
Seldom or never daydreams

２．時々、ボーッとしている
Sometimes daydreams

３．度々、ボーッとしている
Frequently daydreams
